# Impact of Modified Atmosphere Packaging Conditions on Quality of Dates: Experimental Study and Predictive Analysis Using Artificial Neural Networks

**DOI:** 10.3390/foods12203811

**Published:** 2023-10-17

**Authors:** Abdelrahman R. Ahmed, Salah M. Aleid, Maged Mohammed

**Affiliations:** 1 Department of Food and Nutrition Sciences, College of Agricultural and Food Sciences, King Faisal University, P.O. Box 400, Al-Ahsa 31982, Saudi Arabia; arahmed@kfu.edu.sa (A.R.A.); seid@kfu.edu.sa (S.M.A.); 2 Home Economics Department, Faculty of Specific Education, Ain Shams University, Cairo 11566, Egypt; 3 Date Palm Research Center of Excellence, King Faisal University, Al-Ahsa 31982, Saudi Arabia; 4 Department of Agricultural and Biosystems Engineering, Faculty of Agriculture, Menoufia University, Shebin El Koum 32514, Egypt

**Keywords:** date characteristics, machine learning (ML), prediction models, food preservation, food quality, food supply chain, storage conditions

## Abstract

Dates are highly perishable fruits, and maintaining their quality during storage is crucial. The current study aims to investigate the impact of storage conditions on the quality of dates (Khalas and Sukary cultivars) at the Tamer stage and predict their quality attributes during storage using artificial neural networks (ANN). The studied storage conditions were the modified atmosphere packing (MAP) gases (CO_2_, O_2_, and N), packaging materials, storage temperature, and storage time, and the evaluated quality attributes were moisture content, firmness, color parameters (L*, a*, b*, and ∆E), pH, water activity, total soluble solids, and microbial contamination. The findings demonstrated that the storage conditions significantly impacted (*p* < 0.05) the quality of the two stored date cultivars. The use of MAP with 20% CO_2_ + 80% N had a high potential to decrease the rate of color transformation and microbial growth of dates stored at 4 °C for both stored date cultivars. The developed ANN models efficiently predicted the quality changes of stored dates closely aligned with observed values under the different storage conditions, as evidenced by low Root Mean Square Error (RMSE) and Mean Absolute Percentage Error (MAPE) values. In addition, the reliability of the developed ANN models was further affirmed by the linear regression between predicted and measured values, which closely follow the 1:1 line, with R^2^ values ranging from 0.766 to 0.980, the ANN models demonstrate accurate estimating of fruit quality attributes. The study’s findings contribute to food quality and supply chain management through the identification of optimal storage conditions and predicting the fruit quality during storage under different atmosphere conditions, thereby minimizing food waste and enhancing food safety.

## 1. Introduction

Date palms (*Phoenix dactylifera* L.) are a major crop in hot, dry regions around the world. They are grown on over 1.3 million hectares and produce nearly 10 million tons of fruit each year. Arab countries account for 70% of the world’s date palms and 67% of global production [1]. The global date market, dominated by countries such as Egypt, Iran, Saudi Arabia, Iraq, and the UAE, is gaining popularity due to its nutritional value and health benefits. Despite the high volume of domestic date production in Saudi Arabia, there is a need to strengthen the domestic market and further expand exports, which have not met anticipated levels [2]. 

Carbohydrates in date fruits, such as soluble sugars (glucose, fructose, sucrose) and dietary fiber (cellulose, hemicelluloses, pectin), are the most important components [3]. In addition, date fruits contain various polyphenols, including phenolic acids, hydroxyl cinnamates, flavonoid glycosides including quercetin, lutein, and pro-anthocyanidin oligomers [4]. Bioactive compounds are naturally present in date palm fruits that comprise varied secondary metabolites. Phenolic acids, carotenoids, and flavonoids were detected. Phenolic acids are the most abundant bioactive components, which are considered the major contributors to the antioxidant activity in date fruits [5]. There is rigorous evidence that date extract is a strong scavenger of free radicals, which support the fact that including dates into the daily human diet led to the health benefits through preventing or alleviating chronic degenerative diseases [6]. Eid et al. [7] studied the beneficial bacteria changes that occur in the intestine when taking whole date fruit extract. The results were taken based on fecal cultures regulated by pH and mimicking the human large intestine. The fluorescence microscopic count indicated a clear increase in bifidobacterial growth within 24 h. The production of flavonoid aglycones (myricetin, luteolin, quercetin and apigenin) was also observed in less than an hour, due to bacterial metabolism. Dates also inhibit pathogens and raise the production of lactate and acetate, which contributes to the promotion of colon health [8]. In addition, the intake of flavonoids can also help to regulate the composition and function of the gut microbiota. This suggests that there is a two-way relationship between flavonoids and gut microbiota, with both influencing each other’s beneficial effects on human health [9]. Recent research has shown that eating flavonoids may protect the brain in a variety of ways. Some flavonoids can cross the blood–brain barrier and enter the central nervous system, where they can help to reduce inflammation and oxidative stress [10].

Physiological, biochemical, and structural changes occur during the storage of date fruits affecting postharvest life and quality, which include enzymatic browning, moisture loss, texture softening, and change in carbohydrate composition [11]. Date fruits are attacked by fungi and bacteria, resulting in a loss of economic output. There is little or no treatment applied to dates offered on the Saudi market to reduce microbial contamination, which could spread during transportation, handling, and storage [12].

Regarding contemporary food analysis, food metabolomics—which primarily assesses food ingredients, quality, processing, and pathogens from farm to table—has been used in food safety control (in the assessment of microbial toxins, allergens, anti-nutritional, food borne pathogens, and pesticides), food quality (organoleptic properties and nutritional value), food authenticity (adulterations and geographic origin), and food traceability [13]. Foodborne pathogens that contaminate foods at any stage from food production to consumption can survive and grow despite the best efforts of food handlers due to the adaptation mechanisms of pathogens, i.e., spore formation, thermos stable bacterial toxins and mycotoxins, and biofilm production [14]. The flavor, texture, or look of food cannot be significantly altered by foodborne infections and/or their toxins, which is more essential. Following numerous foodborne outbreaks highlighting the risks of foodborne diseases, microbial-related contaminants have emerged as the most frequently reported foodborne causative agents [15]. As a result, technologies to identify food pathogens and bio-toxins quickly and sensitively have been developed and implemented. Metabolomics is a powerful tool for studying the small molecules, called metabolites, that are found in living organisms. Metabolites play a vital role in all aspects of cell function, and their levels can change in response to environmental factors, such as storage conditions. Metabolomics can be used to develop new and improved methods for assessing the quality of dates during postharvest storage. This is accomplished by identifying and quantifying key metabolites that are associated with date quality, such as sugars, acids, and antioxidants. These metabolites can then be used to develop biomarkers that can be used to predict the shelf life of dates and to identify dates that are of poor quality. One challenge of using metabolomics to assess date quality is that the metabolome of dates is complex and dynamic. This means that it is difficult to identify all of the metabolites that are associated with date quality. By developing new and improved methods for predicting the shelf life of dates and for identifying dates that are of poor quality, metabolomics can help to reduce food waste and to ensure that consumers have access to high-quality dates [16].

There are certain techniques to protect stored fruits; modified atmosphere packaging (MAP) is one of the most important techniques that could be used to extend the shelf life of fresh foods. MAP technology provides an environment with low O_2_ and high CO_2_ levels that can impede oxidation reactions, microbial spoilage of food, and food shrinkage and improve sensory quality compared to a normal atmosphere. Consumers are increasingly accepting MAP technology as it continues to develop. Therefore, the MAP obtained a common technique used in the food industry to prolong the shelf life of fresh produce. Modifying the air composition inside the package will create an optimal environment for stored fruits to remain fresh longer [17]. Good flavor and characteristics of fresh foods are ensured by controlling the gas composition and temperature, which is critical [18]. Employing MAP can delay surface browning and maintain the quality of litchi at low temperatures (5 ± 1 °C) by preventing the activities of polyphenol oxidase and peroxidase [19].

Despite great efforts to maintain the quality and safety of date palms, fungi, bacteria, and insects can attack both pre- and post-harvested date fruits, resulting in economic loss. In Saudi Arabia, most dates are consumed without any treatment to reduce microbial contamination, which increases the microbial load during transportation, handling, and storage. The high sugars and moisture content levels, coupled with humid weather during harvesting and ripening, make date fruits susceptible to fungal infections [20]. Bacteria, fungi, and yeasts are the primary causes of date spoilage during storage. To combat this issue, MAP technique is used to modify the air composition, creating an ideal atmosphere that extends the date’s storage life and quality [21]. During storage, respiration results in O_2_ consumption and CO_2_ production. Therefore, O_2_ concentrations are usually decreased to reduce microbial activity, and CO_2_ concentrations are increased above atmospheric levels [22]. This reduces the growth of gram-negative and aerobic spoilage organisms [23]. In this study, MAP technique was combined with cold storage temperatures to enhance the safety and shelf life of whole date fruits.

Predictive analysis using artificial neural networks (ANN) is a technique that uses machine learning (ML) algorithms to analyze historical data and make predictions about outcomes. ANN is a deep learning model inspired by the human brain’s structure and function. It consists of multiple interconnected layers of artificial neurons, known as nodes or units, which process information and make predictions based on the patterns in the input data [24,25]. ANN consists of interconnected nodes resembling biological neurons. The links between the nodes, like synapses in the brain, can send signals to other neurons. ANN offers adaptive learning, where the learning rate determines the size of the model’s corrective steps in adjusting for errors. A high learning rate reduces training time but reduces accuracy, while a low learning rate takes longer but offers the potential for greater accuracy. The performance of ANNs in complex problems depends strongly on their networks architecture for the best solutions. Neural networks are beneficial for handling large datasets and can process continuous data streams faster than linear models. Therefore, ANNs were used in this study due to their ability to model complex physics problems to obtain optimal solutions [26,27]. Therefore, ANNs are computational models that can learn from data and predict outcomes for complex problems [28]. ANNs can identify patterns and relationships in large datasets. ANNs can capture the nonlinear relationships between input and output variables without requiring prior assumptions or explicit equations. ANNs differ from other modeling techniques like multi-linear regression because they can use multiple variables to predict various parameters. They can also learn about the process they represent without knowing the input or output variables. This makes ANNs useful tools for food quality and safety, such as modeling microbial growth; analyzing spectroscopic data; and predicting food products’ food safety and physiochemical, sensory, and functional properties during processing, storage, and distribution. Moreover, ANNs are an innovative technique with more potential for complex modeling tasks in simulation and process control for food quality and safety management [24]. ANN has been widely used for modeling and optimizing various aspects of food processing and quality [29]. For example, ANN has been used to model the effect of different factors on the quality of fruits and vegetables [27,30].

Although MAP has been successfully used for many fruits and vegetables, its impact on the quality characteristics of dates during storage is still not fully understood. Moreover, storage time is an important factor affecting the dates’ quality. Therefore, it is essential to investigate and model the effect of MAP and storage time on the quality characteristics of dates to determine the optimal conditions for their storage and preservation. This study presents a comprehensive investigation into the postharvest storage of date fruits, focusing on the utilization of Artificial Neural Network (ANN) models to predict and optimize key quality attributes. While previous research has studied various aspects of date fruit preservation, this study uniquely combines the ANN modeling with extensive experimental data to provide accurate predictions of moisture content, water activity, pH, total soluble solids, and microbiological load. The innovative aspect lies in the development of predictive models tailored to specific date cultivars, Khalas and Sukary, which have distinct characteristics. By incorporating ANN models, this research offers a practical approach to enhance storage conditions, reduce quality deterioration, and minimize product losses. Therefore, the study aimed to investigate the influence of controlling factors on stored dates, such as providing an environment with low O_2_ and high CO_2_ levels, storage temperature, and package type as measures that can preserve dates’ freshness, limit microbial spoilage, and improve keeping quality and in addition, developing ANN models that can predict the impact of MAP and storage conditions on the quality of date palm fruits, i.e., moisture content, water activity, total soluble solids, pH, color, and microbial load to provide useful information for date fruit producers for optimizing the MAP parameters and storage conditions for reducing food waste and enhance food safety.

## 2. Materials and Methods

### 2.1. Material and Samples Preparation

Khalas and Sukary date samples at ‘Tamr’ stage were obtained from a local date farm in Alahsa, Saudi Arabia. All date fruits were pre-cooled and randomly separated at least 500 g into unsealed cardboard boxes or sealed single layer film modified air trays with a dimension of 37 × 137 × 187 mm from VC999 Packaging Systems AG (Melonenstrasse 2, CH—9100 Herisau, Switzerland).

The sealing film (VC999 Packing Systems AG, Herisau, Switzerland) with high barrier selectively permeable was used. The film was tested for water vapor transmission rate (WVTR), oxygen transmission rate (O_2_TR) and carbon dioxide transmission rate (CO_2_TR) at the School of Packaging labs, Michigan State University. The film was tested using a 50 cm^2^ test area of film and run in duplicate. The measured WVTR, OTR, and CO_2_TR results for the tested packaging films are provided in (Table 1).

Table 2 presents the treatment conditions and their codes for two date palm fruit cultivars. The target MAP gas mixture concentrations were introduced from a premixed cylinder, as shown in Table 2.

The gas concentration levels were validated in the sealed MAP trays immediately after completion of tray packaging with the target gases and during storage time, using the gas analyzer device (OXYBABY^®^ M+ gas, Gase Technik GmbH & Co KG, Salinger Feld, Witten, Germany).

### 2.2. Dates Quality Measurements

The quality parameters of date palm cultivars (Khalas and Sukary date) with two methods of packaging (Cardboard box and MAP) stored at 24 °C and 4 °C temperatures were performed for all 12 treatments as mentioned above in Table 1 during storage at 0, 2, 4, 6, 8, 10, and 12 months. The parameters were moisture content (MC), pH, water activity (aw), total soluble solids (TSS), color, and microbial load. All analysis was made with three replicates.

#### 2.2.1. Moisture Content

Moisture content (MC) was determined by the Official Method of Analysis of the Association of Analytical Chemists (AOAC) [31] using a portable electronic moisture balance (Model MOC-120H, Shimadzu Corporation, Kyoto, Japan). Approximately 5 g of the flesh date sample was used. Samples were oven-dried at 105 ± 1 °C until a constant mass was obtained. The MC % of the date fruit of each sample was determined in triplicate. A completely randomized design of the experiment was performed.

#### 2.2.2. pH

Fruit pH was determined according to AOAC [18]; 10 g of a homogeneous sample was mixed with 100 mL of distilled water and homogenized by stirring. Then, a pH meter (Jenway model 3510, Bibby Scientific Ltd., Stone, UK) was calibrated with buffer solutions at 7 and 4 pH before sample measurement.

#### 2.2.3. Water Activity

Water activity (aw) represents the ratio of the water vapor pressure to the pure water vapor pressure at room temperature in a controlled system. The aw was measured according to AOAC [31] using a LabSwift-aw water activity meter (Novasina AG, Lachen, Germany).

#### 2.2.4. Total Soluble Solids

Total soluble solids (TSS) were determined according to AOAC [31], and 10 g of a homogeneous sample was homogenized by stirring with 10 mL of distilled water. Then, the mixture was filtered using a piece of cloth. Afterward, two drops of the fruit juice were placed in a refractometer (Bellingham + Stanley Limited, 26-860, Wells, UK) lens and the TSS was read as degrees Brix.

#### 2.2.5. Color Parameters

The color parameters of dates before and at each storage time were measured according to the method described by Hunter and Harold [32] using a digital Hunter lab Color Quest-4500 LAV color difference meter (Hunter Associates Laboratory, Inc., Reston, VA, USA) as CIE L, a and b values [33]. In this coordinate system, the L value is a measure of lightness ranging from 0 (black) to 100 (white), a value ranges from −100 (greenness) to +100 (redness), and the b value ranges from −100 (blueness) to +100 (yellowness). As the values of a* and b* rise, the color becomes more chromatic or saturated. These values approach zero for neutral colors (white, grey, black). The instrument was calibrated with a white primary tile supplied by the manufacturer. Twelve readings were taken on each sample. After eliminating the two extreme readings, the remaining ten were averaged and recorded with their standard deviation. The color difference (∆E) was calculated according to International Commission on Illumination (CIE) lab using the following formula:

(1) $$\Delta E^{*}=\sqrt{{\left(L_{i}^{*}-L_{s}^{*}\right)}^{2}+{\left(a_{i}^{*}-a_{s}^{*}\right)}^{2}+{\left(b_{i}^{*}-b_{s}^{*}\right)}^{2}}\;$$

where ∆E is the color difference of the fruit, L* is the lightness, a* is the greenness–redness, b* is the blueness–yellowness, iis the initial value, and s is the value after target storage time.

#### 2.2.6. Microbiological Load

Samples (10 g) were weighed into sterile stomacher bags, 90 mL sterile peptone water (CM0009; Oxoid, Basingstoke, UK) added, homogenized in a stomacher (Lab-Blender 400; Seward Medical, Worthing, UK) for 45 s and aliquots (1.0 or 0.1 mL) plated out in duplicate as 10-fold dilutions in peptone water. Aerobic mesophilic bacteria were counted on plate count agar dishes (CM0325; PCA Oxoid) incubated at 30 °C for 2–3 days, yeasts on potato dextrose agar plates (CM0139; PDA Oxoid) at 30 °C for 3 days and molds on potato dextrose agar plates at 20–30 °C for 3–7 days [34].

### 2.3. Artificial Neural Networks

In this study, artificial neural networks (ANN) algorithms have been used as an alternative approach to predict date fruit quality attributes during storage using SPSS version 26.

**Figure 1:**
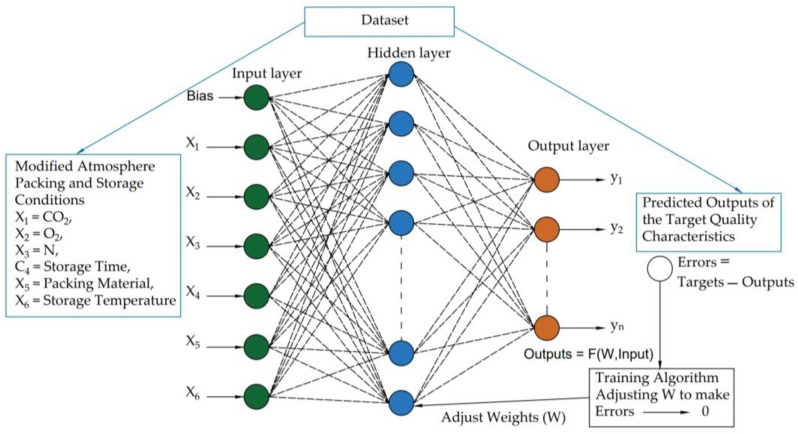
Multi-layer feedforward artificial neural network structure.

A multi-layer feedforward ANN structure consists of input, hidden, and output nodes interconnected through weights, as shown in Figure 1. The ANN mimics the structure of the human brain, with each layer comprising multiple nodes that can connect to nodes in other layers. The input layer sends signals to the hidden layers and then to the output layer, where the final output was produced. Activation functions were applied to hidden and output layers, and several activation functions determine the characteristics of the ANN. The ANN’ weights were learned through a process that uses input and target pairs to identify patterns or features in each dataset.

The outputs for a given ANN structure were calculated based on its current weights and inputs from the dataset (outputs = F (W, inputs)). The training signals for adjusting the weights of the ANN via an optimization procedure were formed by comparing the outputs to the targets from the dataset. This process was repeated until the differences between the ANN outputs and corresponding targets, also known as errors, were minimal. As errors were calculated from the output of the ANN layer and used to adjust the weights from hidden layers to the input layer, this process is called back-propagation. Eventually, the ANN will learn the behavior of the dataset through this training process.

To perform predictive models using the ANN, the following steps were performed:

- Dataset preparation: The dataset preparation involved two separate stages. In the initial stage of the experiment, experimental data collection was carried out to train, test, and evaluate the ANN models. During this stage, the dataset was divided to 60% for model training, 20% for testing the model’s performance, and an additional 20% for evaluation. In the second stage, new data, entirely separate from the dataset employed in model development, was utilized for the purpose of model validation. This separation was performed to ensure that the ANN models were validated on unseen data, preventing any potential bias or overfitting that might occur if the same data were used for both training and validation.
- Training the ANN model: in this phase, 60% of the dataset was used to train the ANN model. This involved providing the ANN with input data, which consisted of various storage parameters of storage time, storage temperature, packing material, N, O_2_, and CO_2_. The model learned to map these input variables to the target output variables of stored date fruit quality attributes, i.e., moisture content, water activity, total soluble solids, pH, color parameters, and microbial counts. The ANN adjusted its internal weights and biases during this phase to minimize the sum of square errors between the predicted values and the actual values in the training dataset.
- Testing the ANN: In this phase, 20% of the dataset that was not used during training was set aside for testing the model’s performance. The ANN was used to predict the quality attributes for this testing dataset based on the same conditions. The error between the predicted values and the actual values for this dataset was calculated to assess how well the model generalized to unseen data.
- Evaluation of the ANN models: In this phase, the remaining 20% of the dataset was employed for the evaluation phase, which served as an independent validation dataset. Again, the ANN was used to predict the quality attributes for this dataset. The error metrics of RMSE and MAPE were calculated for the evaluation of the unseen dataset. This phase was conducted to evaluate the performance of the trained ANN model to assess how well the developed model predicts future outcomes.
- Validation: The validation of the implemented ANN prediction model was conducted after evaluating the developed models on the separate data. By providing the new input data to the trained networks, the model generated predictions based on the patterns it learned during the training phase. The linear regression analysis was performed to compare the predicted values with the observed values for the separated data of various quality parameters to validate the models.

### 2.4. Statistical Analysis and ANN Evaluation

The experimental design in this study included two phases. In the first phase, data collection was performed to investigate the impact of MAP conditions during storage on the quality of stored date fruits using analysis of variance (ANOVA) using SPSS version 26 (SPSS Inc., Chicago, IL, USA) by four analyses during the storage period (0, 2, 4, 6, 8, 10, and 12 months) of the stored date cultivars under the different storage conditions of KCaRT, KCaCT, KNAPRT, KNAPCT, KMAPRT, KMAPCT, SCaRT, SCaCT, SNAPRT, SNAPCT, SMAPRT, and SMAPCT. Duncan’s Multiple Range Test (MRT) was used to determine the significant variance levels between treatments within the level of significance (*p* < 0.05). Within this stage, three replicates were conducted for each of the three different replicated samples of each treatment. Each replicate value was determined as the average of five measurements, each derived from five different fruits obtained from the same samples. This procedure yielded a total of 15 measurements for each treatment.

For training, testing, and evaluating the ANN models, the collected data in the first phase were prepared. Each quality attribute of the date fruit cultivars was based on a total of 630 values for each cultivar. This dataset size was considered suitable for ensuring the models’ accuracy in predicting each fruit quality attribute for each fruit cultivar.

In the second phase, an additional set of 252 new measurements was used entirely separately from the dataset used for model development. Linear regression analyses were conducted, comparing the predicted values generated by the models with the observed values derived from this new dataset of 252 measurements for each quality attribute to substantiate the reliability and precision of the ANN models in their predictions of fruit quality attributes.

The results were statistically analyzed through the analysis of variance (ANOVA) using SPSS version 26 by four analyses during the storage period (0, 2, 4, 6, 8, 10, and 12 months) of the stored date cultivars under the different storage conditions of KCaRT, KCaCT, KNAPRT, KNAPCT, KMAPRT, KMAPCT, SCaRT, SCaCT, SNAPRT, SNAPCT, SMAPRT, and SMAPCT by randomized complete block design. Duncan’s Multiple Range Test (MRT) was used to determine the significant variance levels between treatments within the level of significance (*p* < 0.05).

The relative error, coefficient of determination values, mean absolute percentage error, and root mean square error were used to evaluate the developed ANN models. The formulas of these evaluation metrics were as follows:

(2) $$RE=\frac{\left|O_{i}-P_{i}\right|}{O_{i}}$$

(3) $$R^{2}=1-\frac{\sum {\left(O_{i}-P_{i}\right)}^{2}}{\sum O_{i}^{2}-\frac{{\left(\sum O_{i}\right)}^{2}}{n}}$$

(4) $$MAPE=100\times \frac{1}{n}\times \sum_{i=1}^{n}\left|\frac{O_{i}-P_{i}}{O_{i}}\right|$$

(5) $$RMSE=\sqrt{\frac{\sum_{i=1}^{n}{\left(O_{i}-P_{i}\right)}^{2}}{n}}$$

where RE is the relative error, R^2^ is the coefficient of determination, mean absolute percentage error is MAPE, and RMSE is root mean square error, $\left|O_{i}-P_{i}\right|$ is the absolute error, O_i_ is the measured/observed value, n is the number of the measure values, and P_i_ is the predicted values of the target parameter data i.

## 3. Results and Discussion

### 3.1. Changes in Gas Concentrations

The changes in O_2_ and CO_2_ levels of date fruit packages were followed for one year every 10 days. The atmosphere around the KCaRT and SCaRT samples in the storage room with the controlled temperature of 24 ± 1 °C and relative humidity of 36 ± 5.4% RH was normal air (20.95% O_2_ and 0.03% CO_2_). The concentration of the O_2_ ranged from 20.95 to 20.25%, and CO_2_ ranged from 0.03 to 0.05% in the atmosphere around the KCaCT and SCaCT samples in the cold storage room with a controlled temperature of 4 ± 0.5 °C and relative humidity of 65 ± 2% RH. In SNAPRT treatments, the O_2_ was reduced from 20.9 to 5.2%, and the CO_2_ increased from 0.03 to 10.8% after one month. Then, O_2_ levels decreased slightly, while CO_2_ levels increased from 10.8 to 14.9%, remaining almost stable until the end of the storage period. In KNAPRT treatments, the O_2_ was reduced from 20.9 to 12.4%, and the CO_2_ increased from 0.03 to 6.9% after one month. Then, O_2_ levels decreased slightly, while CO_2_ levels increased from 6.9 to 10.7%, remaining almost stable until the end of the storage period. In SNAPCT treatments, the O_2_ was reduced from 20.9 to 14.2%, and the CO_2_ increased from 0.03 to 6.3% after one month. Then, O_2_ levels decreased slightly, while CO_2_ levels increased from 6.3 to 8.7%, remaining almost stable until the end of the storage period. In KNAPRT treatments, the O_2_ reduced from 20.9 to 16.9%, and the CO_2_ increased from 0.03 to 3.8% after one month. Then, O_2_ levels decreased slightly, while CO_2_ levels increased from 3.8 to 5.9%, remaining almost stable until the end of the storage period. The concentration of CO_2_ in the KMAPRT, SMAPRT, KMAPCT, and SMAPCT treatments was almost the same. In KMAPRT and SMAPRT, rich in CO_2_ with zero O_2_, the CO_2_ level gradually decreased from 20% to 14.3% after one month. In KMAPRT and SMAPRT, the CO_2_ level slowly decreased from 20% to 16.3% and the CO_2_ from 20% to 12% after one month. It is recognized that steady-state O_2_ and CO_2_ concentrations inside a package depend on fruit respiration rate and film permeability [35].

### 3.2. Quality of Date Fruits Data

#### 3.2.1. Chemical Properties

Table 3 presents the mean values of the chemical properties of the studied date cultivars under different storage conditions. The letters in this table indicate whether there are significant differences at *p* ≤ 0.05 between the means of different storage conditions for each date cultivar. The table shows that there were significant differences at *p* ≤ 0.05 in the mean values of each parameter across different storage conditions that indicate that the storage conditions impact the quality parameters of the date cultivars.

For the Khalas cultivar, the mean values of MC were varied significantly across different storage treatments, with the lowest value observed in KCaRT (14.14%) and the highest value in KCaCT (18.48%). The mean values of aw also show significant differences between storage conditions, ranging from 0.41 in KCaRT to 0.59 in KMAPCT. The mean values of TSS vary significantly, with the lowest value in KCaRT (62.59 Brix) and the highest value in KCaCT (65.79 Brix). The mean pH values also exhibit significant differences between storage conditions, ranging from 5.51 in KMAPCT to 5.78 in KCaRT.

For the Sukary cultivar, the mean values of MC show significant differences between storage conditions, with the lowest value observed in SCaRT (9.64%) and the highest value in SCaCT (15.16%). The mean values of aw also exhibit significant differences, ranging from 0.42 in SCaRT to 0.64 in SCaCT. The mean values of TSS vary significantly, with the lowest value in SCaRT (62.95 Brix) and the highest value in SCaCT (68.22 Brix). The mean pH values show significant differences between storage conditions, ranging from 5.42 in SMAPCT to 5.79 in SCaRT.

**Figure 2:**
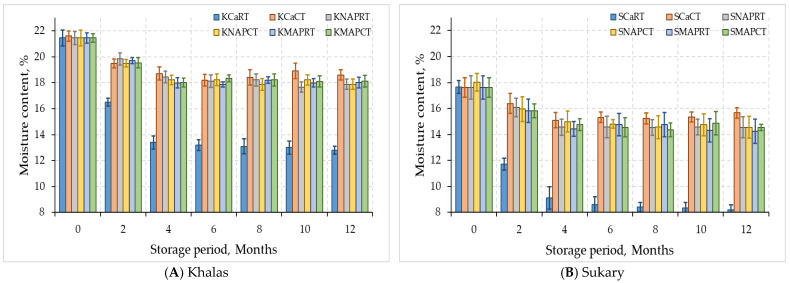
Moisture contents for MAP-treated dates Khalas (**A**) and Sukary (**B**) cultivars stored under different packaging and temperatures.

Figure 2 shows the changes in moisture content of Khalas and Sukary cultivars throughout the storage period. The stored Khalas and Sukary fruits experienced alterations in their moisture content and water activity over 12 months of refrigeration (4 °C) and room temperature storage (24 °C). Date palm fruits lose moisture rapidly during the ripening process in the traditional storage in room temperature. Therefore, the moisture content under treatments of KCaRT and SCaRT significantly decreased (*p* < 0.05) by 33.4% and 46.1%, respectively, while a slight reduction in the fruit moisture content has been observed under the other treatments after 12 months compared to zero time, as shown in Figure 2.

**Figure 3:**
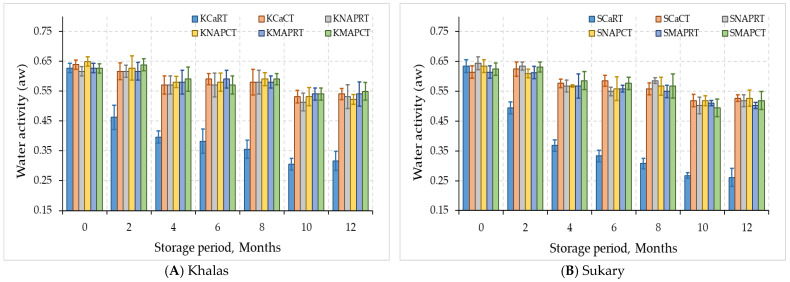
Water activity (aw) for MAP-treated dates Khalas (**A**) and Sukary (**B**) cultivars stored under different packaging and temperatures.

Figure 3 shows the changes in the water activity of Khalas and Sukary cultivars throughout the storage period. The stored Khalas and Sukary fruits experienced alterations in their moisture content and water activity over 12 months of refrigeration (4 °C) and room temperature storage (24 °C). The water activity under treatments of KCaRT and SCaRT significantly decreased (*p* < 0.05) by 50.7% and 69.3%, respectively, while a slight reduction in the water activity occurred under the other treatments after 12 months compared to zero time, as shown in Figure 2.

The reduction in moisture and water activity under KCaRT and SCaRT treatments were attributed to the evaporation of water from the dates caused by the relatively high temperature and moderate relative humidity. Date fruits of various date palm cultivars have a moisture level of less than 30%, which decreases to less than 24% at the Tamar stage and eventually reaches 4–10% in fully ripened dry date cultivars [28]. Previous studies have reported changes in the moisture content of date fruits. Ihsanullah et al. [36] found that the moisture content of date fruits packed in white polythene decreased from 14.1% to 9.7% over five months. Khan et al. [37] reported that the moisture content of dry date fruits decreased from 14.4% to 12.8% after twelve months of storage at ambient temperature. Other studies have shown that fruit moisture levels tend to remain stable during storage under low temperatures, but extended storage periods can reduce moisture content [38].

Considering both MAP trays treatments either with CO_2_ + N or normal air, both cultivars showed no significant difference (*p* > 0.05) in moisture content and water activity at cold temperatures compared to control during storage period 12 months. Water activity of date fruits played a vital role in governing pH changes and samples with reduced water activity displayed greater resistance against date fruits deterioration [39]. The moisture content and water activity percentage after 6 months of KNAPCT and SNAPCT dates packed in natural air were 17.88, 14.20% and 0.59, 0.60, respectively. While with KMAPCT and SMAPCT, which were packed in CO_2_ + N, it was 18.11, 14.54%, for moisture content and 0.61, 0.64 for water activity after 12 months of storage, respectively. It was observed that if the storage time was slightly increased, the moisture loss also increased in the samples packed in natural air. This difference might be due to the respiration of the stored dates.

Generally, at all the storage times in the natural air packaging at room temperature for Khalas and Sukary, there was a low significant reduction in moisture losses, remaining within acceptable limits. The slight decrease in the moisture content and water activity of the stored dates in the cartons was because the cold storage is equipped with an automatic humidification system designed by Mohammed et al. [40,41]. The humidification system helps maintain the ideal humidity level to prevent the dates from becoming too dry or too moist, thus ensuring their quality during storage. As shown in Figure 2, there was a relation between the storage via MAP and the least moisture loss in the dates. This was mainly attributed to moisture loss under air packaging conditions, while MAP-treated dates were enclosed in packages that prevented the loss of excess water content [42]. Al-Redhaiman [43] reported that the weight loss of Barhi dates was an inversely proportional relationship between CO_2_ concentration at storing containers and the weight loss percent of the fruit. The highest percentage of moisture loss of the fruit was observed in the control in a cardboard box, followed by packing in normal air, whereas the lowest percentage of the fruit moisture loss was recorded for the fruits that were packed in a 20% CO_2_ gas mixture.

**Figure 4:**
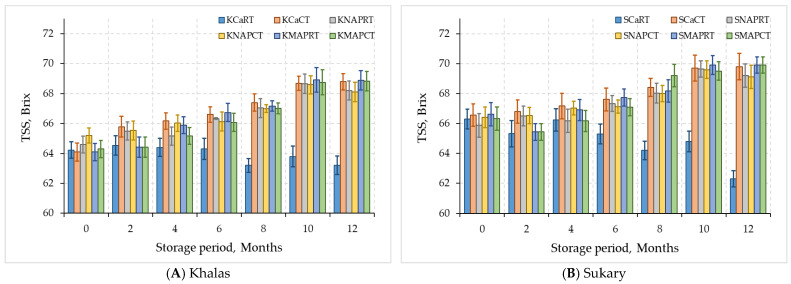
TSS for MAP-treated dates Khalas (**A**) and Sukary (**B**) cultivars stored under different packaging and temperatures.

The total soluble solids (TSS) were strongly associated with the perception of sweetness, date flavor, and aroma intensity. The sugars present in the flesh of dates primarily include fructose, glucose, and sucrose. The sugars are found in varying proportions among different cultivars at the maturation level [44]. The TSS of Khalas and Sukary fruits that were stored at refrigeration (4 °C) and room temperature for 12 months changed (Figure 4). KCaRT and SCaRT showed a significant decrease (*p* < 0.05) in TSS, while the stored fruits under the other treatments have a significant increase in their TSS contents after 12 months compared to the initial time, as shown in Figure 4. This increase could be attributed to the conversion of insoluble compounds into soluble compounds, such as the conversion of proto pectin into pectin, in the date samples. Significant differences (*p* < 0.05) in TSS were observed between the two cultivars under different packing types. These findings were consistent with those of Lee et al. [45] and Azelmat et al. [46] who reported a gradual increase in TSS with storage time due to the degradation of insoluble compounds present in the date fruit. El-Gioushy et al. [47] reported increased TSS for Barhi date palm cultivar stored for 70 days at 0 °C and 90–95% RH. Similarly, Akhavan et al. [48] observed increased TSS for Mazafati date palm cultivar fruits stored for 180 days at 4 °C. The slow increases in TSS in MAP date fruit may have been due to the addition of altered gas atmospheres or the optimal storage temperature [49,50]. The enzymatic conversion of large polysaccharides into small sugars was likely the primary reason for the increase in TSS [51]. Radi et al. [52] suggested that the increase in TSS during storage could be due to microbial and enzymatic activities that degrade high molecular weight compounds to low molecular-weight ones.

**Figure 5:**
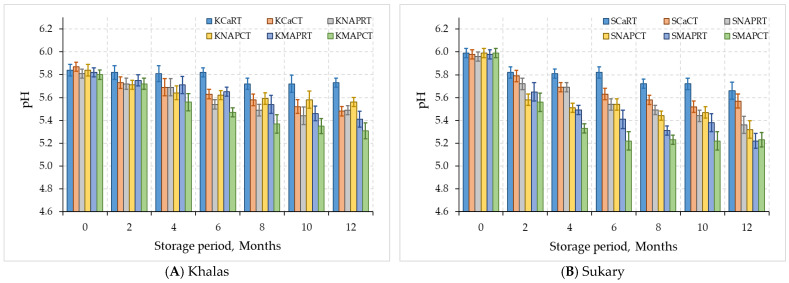
pH for MAP-treated dates Khalas (**A**) and Sukary (**B**) cultivars stored under different packaging and temperatures.

The pH of dates samples significantly decreased as the storage period increased, particularly in samples stored at 24 °C compared to those stored at 4 °C (Figure 5). Significant differences (*p* < 0.05) in pH were observed between the two cultivars under different packing types. This could be due to the higher metabolic activity of microorganisms at higher temperatures [53]. The pH of KMAPCT dates decreased from 5.80 to 5.31, while the pH of SMAPCT dates decreased from 5.95 to 5.23 during the storage period of 12 months. Similar findings were reported in a study on Dhakki dates, Baloch et al. [39] observed a gradual decline in pH during storage. The continuous decrease in pH during storage could be attributed to oxidative and non-oxidative mechanisms. The difference in pH values between MAP samples was significant (*p* < 0.05) after 12 months of storage [40,54], with the pH reduction mostly due to CO_2_ solubility in the fruits’ flesh. The temperature increase had a more significant effect on pH reduction than CO_2_ elevation. The pH variations could affect the fruits’ flavor, aroma, texture, and shelf life due to organic acids, which vary from cultivar to cultivar. The study found that all date samples had pH levels greater than 4.6, indicating low-acidic values. The fruit pH decreases with maturity stages, with the lowest pH observed at the final stage of ripening [55,56,57].

Dates, like all living organisms, continue to respire even after being harvested. During postharvest storage, dates undergo several metabolic changes, including respiration, which consume O_2_ and produce CO_2_, water, and energy. The metabolic changes in date fruits during storage are important processes that affect their quality attributes. The metabolic changes occur because of various biochemical processes and can significantly impact the quality attributes and shelf life of the fruit. This metabolic process is essential to sustain the fruit’s physiological functions, but it can also lead to the production of undesirable byproducts that affect fruit quality. In addition, dates are naturally rich in sugars, primarily glucose, and fructose. Sugar metabolism plays a central role during storage. As dates respire, they may convert some of their sugars into other compounds, such as organic acids, which can influence taste and acidity levels. Additionally, changes in sugar content can affect TSS and texture, which are critical quality attributes. Organic acids, such as citric and malic acid, are involved in the regulation of pH in date fruits. Metabolic changes in organic acid levels can impact the overall acidity and tartness of the fruit. Although aw is not a metabolic change per se, water activity (aw) is a critical factor affecting the metabolic processes in dates. As aw decreases during storage, metabolic activities slow down, which can contribute to extended shelf life and reduced susceptibility to spoilage [58,59]. Metabolomics of dates reveals a highly dynamic ripening process accounting for major variation in fruit composition. Three distinct metabolic phases corresponding to known stages of date ripening were observed. An early phase enriched in regulatory hormones, amines and polyamines, energy production, tannins, sucrose and anti-oxidant activity, a second phase with on-going phenylpropanoid secondary metabolism, gene expression and phospholipid metabolism and a late phase with marked sugar dehydration activity and degradation reactions leading to increased volatile synthesis [60]. The metabolism of date fruits during storage is influenced by a combination of factors, including temperature, moisture content, O_2_, and CO_2_ levels, and storage duration. For example, the high changes in fruit quality in samples stored at 24 °C were due to the effect of temperature on metabolic processes, with higher temperatures accelerating enzymatic and microbial activities. O_2_ and CO_2_ levels also play roles, with controlled MAP affecting metabolic rates., the duration of storage with the availability of oxygen gas led to changes in fruit quality.

#### 3.2.2. Color Parameters

The CIE colors (L*, a* and b* values) of the dates were measured. The total color difference (ΔE*), which represents the value of the total difference in the three coordinates (L*, a* and b*), was calculated. Color assessments were made on three replicates. Table 4 presents the mean values of color parameters for the two date cultivars under different storage conditions. The table shows that the mean values of each color parameter (L*, a*, b*, and ∆E*) significantly (*p* ≤ 0.05) vary depending on the cultivar and storage condition. For the Khalas cultivar, there are significant differences in the mean values of L*, a*, b*, and ∆E* between the storage types KCaRT, KCaCT, KNAPRT, KNAPCT, KMAPRT, and KMAPCT. Similarly, for the Sukary cultivar, there are significant differences in the mean values of L*, a*, b*, and ∆E* between the storage types SCaRT, SCaCT, SNAPRT, SNAPCT, SMAPRT, and SMAPCT.

**Figure 6:**
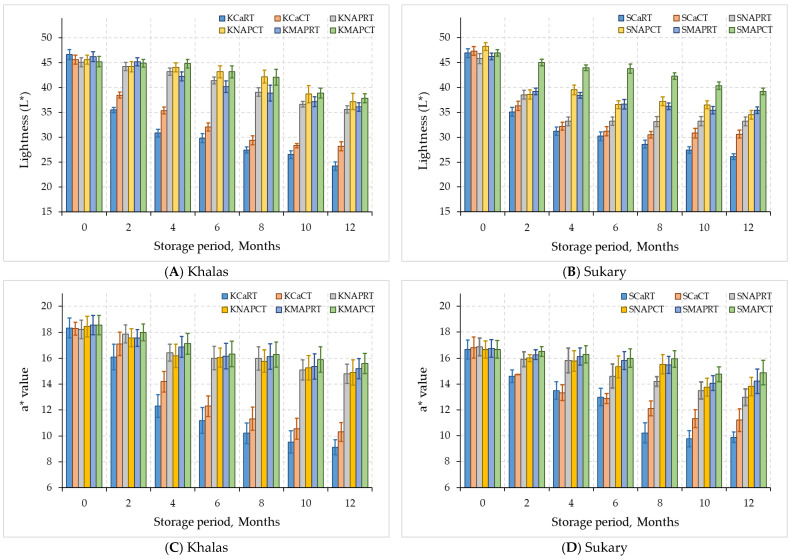

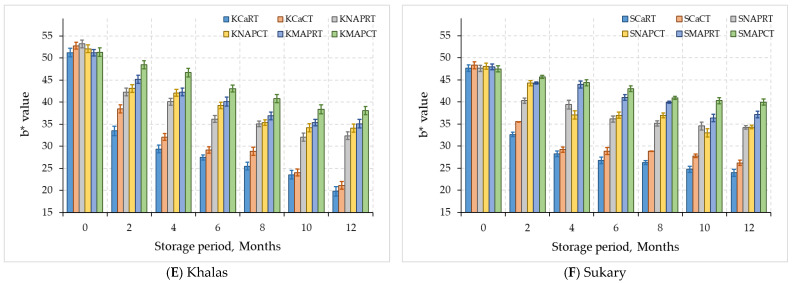
Color parameters of L* (**A**,**B**), a* (**B**,**C**), and b* (**E**,**F**) for MAP-treated date Khalas (**A**,**C**,**E**) and Sukary (**B**,**D**,**F**) cultivars stored under different packaging and temperatures.

Stored ‘Khalas’ and ‘Sukary’ fruits exhibited changes in color all over refrigeration (4 °C) and room temperature (Figure 6). The outer layer of ‘Khalas’ dates is originally light golden brown, while that of ‘Sukary’ dates is a light gold color [11]. In the broad spectrum, ‘Khalas’ fruits become increasingly browner and paler, i.e., lacking glossy intensity, when stored (reflected by a decline in L* and increases in a* and b* values). In a similar manner, ‘Sukary’ fruits lose their bright golden color and become an increasingly dark gold and brown color during storage (Figure 6). The degree of L* values was the highest for the Khalas and Sukary fruits preserved by CO_2_ + N after 10 months of refrigeration as could be seen in KMAPCT and SMAPCT curves, respectively. ‘Khalas’ fruits in this treatment significantly retained their bright golden color, and ‘Sukary’ fruits significantly maintained their light gold expression (high L* and b* values). The control cardboard boxes for both cultivars date fruits either at room temperature or 4 °C for the 12 months presented low L* and high a* and high b* values, representing inferior retention of their original bright color compared to that observed for CO_2_ + N MAP treatments.

Considering both MAP tray treatments either with CO_2_ + N or normal air, both cultivars showed no significant difference in lightness decline with some degree of loss in their initial red and yellow colors at both temperatures. However, the rate of decline was less and moderate in Sukary dates, which shifted into the dull dark brown/cinnamon region (Figure 6), presenting significant differences (*p <* 0.05) from the color appearance associated with the MAP treatments at cold storage at all storage times [61]. Subsequently, at the end of 12 months of storage, all treatments from both cardboard and normal air MAP-treated ‘Khalas and Sukary’ fruits had considerably higher a* and b* values than the control dates, presenting that the fruits were remarkably more red/yellow with less fruit brightness saturation than the control dates.

There is great variation in the color of dates from different date cultivars in Saudi Arabia. Saudi and international standards do not suggest any limits for dates color [62]. Packaging type, storage temperature, and time significantly affected fruit color. In addition, color characteristics may be useful when grading dates according to their colors [63].

**Figure 7:**
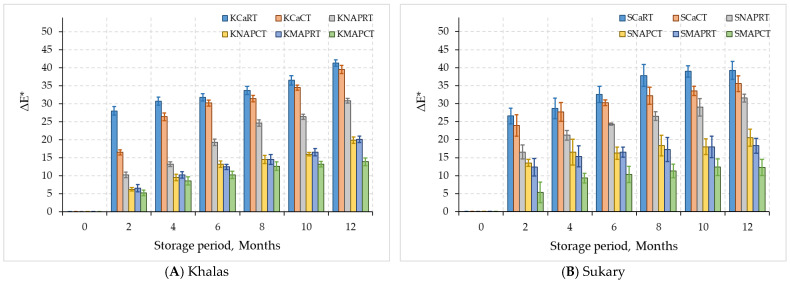
Total color difference (∆E*) for MAP-treated date Khalas (**A**) and Sukary (**B**) cultivars stored under different packaging and temperatures.

The total value of (ΔE*) indicates the overall difference between CO_2_ + N MAP as well as normal air treatments either refrigerated or stored at room temperature and fresh non-treated fruits. The initial (ΔE*) value was zero compared to other trials. The smaller the value was, the better the color safeguarding by the MAP treatment was. Comparing the (ΔE*) values after 2 months’ periods with the initial date samples, both cardboard and MAP tray treatments for ‘Khalas’ and ‘Sukary’ fruits had a considerable increase in ΔE* values (27.2 and 23.3, respectively) (Figure 7). Subsequently, the (ΔE*) values for fruits preserved by CO_2_ + N was almost constant after 10 months of refrigeration for Khalas and 12 months for Sukary. Even though the 0-day control group had the smallest (ΔE*) value, the original pigments in CO_2_+N MAP ‘Khalas’ and ‘Sukary’ fruits from 2 months advancing forward were completely preserved, presenting an acceptable appearance. Kader [64] reported that packing dates in N_2_ decreased fruits darkening. Likewise, Vandercook et al. [65] observed a low browning rate in fresh, O_2_-depleted dates. When the original atmosphere contained 20% CO_2_ + N, the absence of O_2_ in the packaging reduced the accumulation of CO_2_ due to anaerobic respiration throughout refrigeration [42].

#### 3.2.3. Microbial Load

Microbial assessments of the stored ‘Khalas’ and ‘Sukary’ fruits exhibited changes in mesophilic aerobic bacteria, yeast, and mold all over refrigeration and room temperature. Table 5 presents the mean values of microbial loads on the two date cultivars under different storage conditions. The table shows significant differences in the mean values of yeast, molds, and total bacteria depending on the cultivar and storage condition. For example, the mean value of Yeast and Molds for Khalas ranges from 1.66 to 3.27 Log cfu/g, while Sukary ranges from 1.96 to 2.84 Log cfu/g. Similarly, the mean value of total bacteria for Khalas ranges from 1.24 to 2.96 Log cfu/g, while Sukary ranges from 1.61 to 2.19 Log cfu/g. These significant differences in microbial load between the storage conditions for each date cultivar are denoted by different letters in the table, indicating a *p* ≤ 0.05.

**Figure 8:**
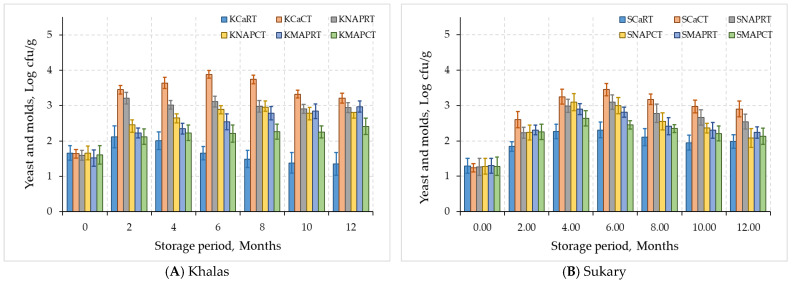

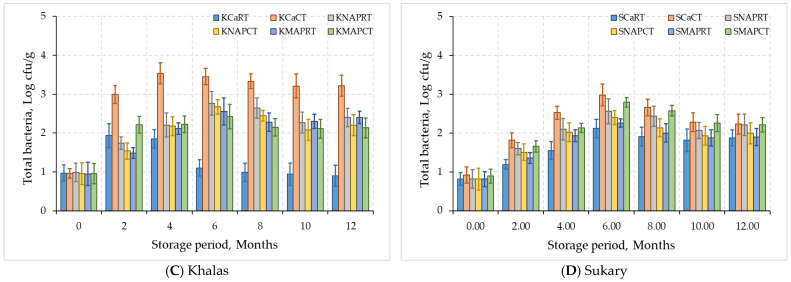
Microbial load: yeast and mold counts (**A**,**B**) and the total bacteria (**B**,**C**) for MAP treated dates Khalas (**A**,**C**) and Sukary (**B**,**D**) cultivars stored under different packaging and temperatures.

The mesophilic aerobic bacteria and yeast and mold counts were the lowest for refrigerated Khalas and Sukary fruits preserved by CO_2_ + N at all storage periods, as seen in KMAPCT and SMAPCT curves, respectively, in Figure 8. Both ‘Khalas’ and ‘Sukary’ fruits significantly scored the highest mesophilic aerobic bacteria and yeast and mold at 6 months of storage for all treatments. However, the highest increase was for the control cardboard boxes (KCaCT) for both cultivars date fruits 4 °C, followed by KNAPRT and SNAPRT treatments at 24 °C compared to CO_2_ + N MAP treatments (KMPCT and SMAPT). There was a slight decline on bacterial and yeast and mold counts at 8 months under all treatments, progressing to the end of 12 months of storage.

Microorganisms, including yeasts, molds, and bacteria, are sensitive to a food’s properties. The aw of dates was one of the most important factors affecting their microbial stability. Dates with higher aw are more susceptible to microbial spoilage as they provide a more favorable environment for the growth of microorganisms. Moreover, low aw of the dates and combination of packaging methods, may have resulted in low microbial load in dates. Preservation methods and low temperature may have prevented microbial proliferation [66]. It was reported that dates have a very high sugar concentration, which gives them a hygroscopic character leading to retain water away from microbial activity. Water activity (aw), derived from water content, is the best parameter used to determine the availability of microbial growth in foods [67].

There is evidence that moisture state is a key factor that influences the composition and activity of microbiota in many environments, including food products, soil, and the gut. Therefore, it is likely that moisture state also plays a role in shaping the microbiota composition of dates. Microorganisms respond to stressful environments, such as low water activity, by preventing cellular damage rather than repairing it [68]. Higher moisture levels create a favorable environment for microbial growth, including bacteria, yeast, and molds. Increased moisture provides the necessary water activity (aw) for microorganisms to thrive and reproduce. Consequently, a higher moisture state in date fruits can lead to an increase in microbial load and diversity, which can negatively impact the fruit’s shelf life and safety. On the other hand, controlling moisture content through appropriate storage conditions can help inhibit the growth of undesirable microorganisms and extend the date fruits’ quality and shelf life. Some of these responses include accumulating osmoprotectants (molecules that help to maintain cell water balance), forming biofilms (communities of microorganisms that are attached to a surface), and growing into long, thin filaments [69]. In an environment with low wa, bacteria must balance the osmolarity (concentration of solutes) inside and outside of their cells to prevent dehydration. To accomplish this, they accumulate Osmo protectants, such as potassium chloride (KCl), glutamate, and trehalose. When bacteria are dried, the resonance of water molecules is less effective due to the low amount of water present [70]. In addition, very low or very high pH values will influence microbial growth. Most bacteria grow in pH 6.5–7.0, while most yeast species thrive at a pH range from 4.5–6.5. When the pH changed; hence, it was optimal for microbial growth on date fruits. It is generally accepted that water activity is more closely related to the physical, chemical, and biological properties of foods and other natural products than its total moisture content. Specific changes in color, aroma, flavor, stability, and acceptability of raw and processed food have been associated with relatively narrow water activity ranges [39]. Variation found within date fruits microbial load could be contributed to pH, water activity, moisture content, storage temperature and duration [71].

Storing dates in a cold temperature and low oxygen atmosphere reduced the microbial growth on the dates and extended their shelf life. The MAP on which date fruits were stored affected their microbial stability. Dates stored in the MAP with a low oxygen content were less likely to spoil than dates stored in an atmosphere with high oxygen content. This is because oxygen is required for the growth of many microorganisms. The current study stored the dates in a prepared atmosphere with a low oxygen content. This helped to reduce microbial growth on the dates [40,72].

In the current study, the water activity of Khalas and Sukary dates significantly decreased in the first four months of storage. Thus, the date samples studied in this study showed high resistance against the growth of microorganisms in this period. The microbial load could be attributed to physicochemical properties, pH, water activity, moisture content, nature and amounts of sugars within dates, external environmental conditions (temperature, humidity), storage conditions, production practices at harvest, and postharvest processing [71]. The current study’s results align with Elhoumaizi et al. [73]. They conducted principal component analysis (PCA) to classify studied ‘Mejhoul’ dates and to provide easy visualization of relationships among their physicochemical and microbiological characteristics. Their results showed that the microbial load of Mejhoul samples is correlated positively to moisture contents [73]. In addition, Jdaini et al. [74] studied the effects of harvesting and postharvest practices on the microbiological quality of dates. They found that total viable counts ranged from 4.2 to 2.6 log cfu/g and yeast/molds reached 2.99 log cfu/g, which can be due to uncontrolled date processes of harvesting and post-harvesting practices. Mohammed et al. [75] found that the average mesophilic aerobic bacteria total counting the samples was 2.09 × 10^4^ cfu/g ranging from 5.85 Log cfu/g and 4.07 Log cfu/g. Zamir et al. [76] explored the microbiological quality of dates and observed that samples to carry 3.30–5.65 Log cfu/g aerobic bacteria and 3.30-5.36 Log cfu/g yeasts and molds. Date samples with no appropriate handling and hygiene practices during postharvest processing were responsible for this contamination. Hamad [77] measured microbial contamination of date fruits immediately after processing and after refrigerated storage at 4 °C for 2, 4 and 6 months. Freshly processed samples were contaminated with mesophilic aerobic bacteria, molds, and yeasts. The amount of contamination decreased steadily with storage time. Aleid et al. [78] found that mesophilic aerobic bacteria, molds, and yeasts loads in packaged dates ranged from 3.29–3.32 and 0.0–2.95 Log cfu/g, respectively.

### 3.3. Correlation between the Characteristics and Storage Parameters

Table 6 presents the correlation between storage parameters and stored fruit of Khalas and Sukary characteristics. This table indicates significant positive and negative correlations (*p* ≤ 0.01) between the storage time, storage temperature, packing material, N, O_2_, and CO_2_ data and the most parameters of fruit quality, i.e., MC, aw, TSS, pH, L*, a*, b*, ∆E, yeast and mold (Y&M), and TAMC. The correlation data presented in the table were important for building and training effective ANN models for predicting the quality attributes of stored dates under MAP. The data allow for the development of a generalized ANN model. Considering data from multiple cultivars (Khalas and Sukary), the model can be trained to capture broader trends and patterns, making it more applicable to different date varieties. The correlation coefficients in the table indicate the strength and direction of relationships between storage parameters and fruit characteristics. The ANN can utilize this information to assign appropriate weights to different features during the learning process, improving the model’s accuracy in predicting the target attributes. The correlation data displayed in the table provide a solid foundation for developing an effective ANN model that can predict the quality attributes of stored dates under MAP.

### 3.4. Predictive Analysis Using ANN

#### 3.4.1. Architecture of the ANN Prediction Models

**Figure 9:**
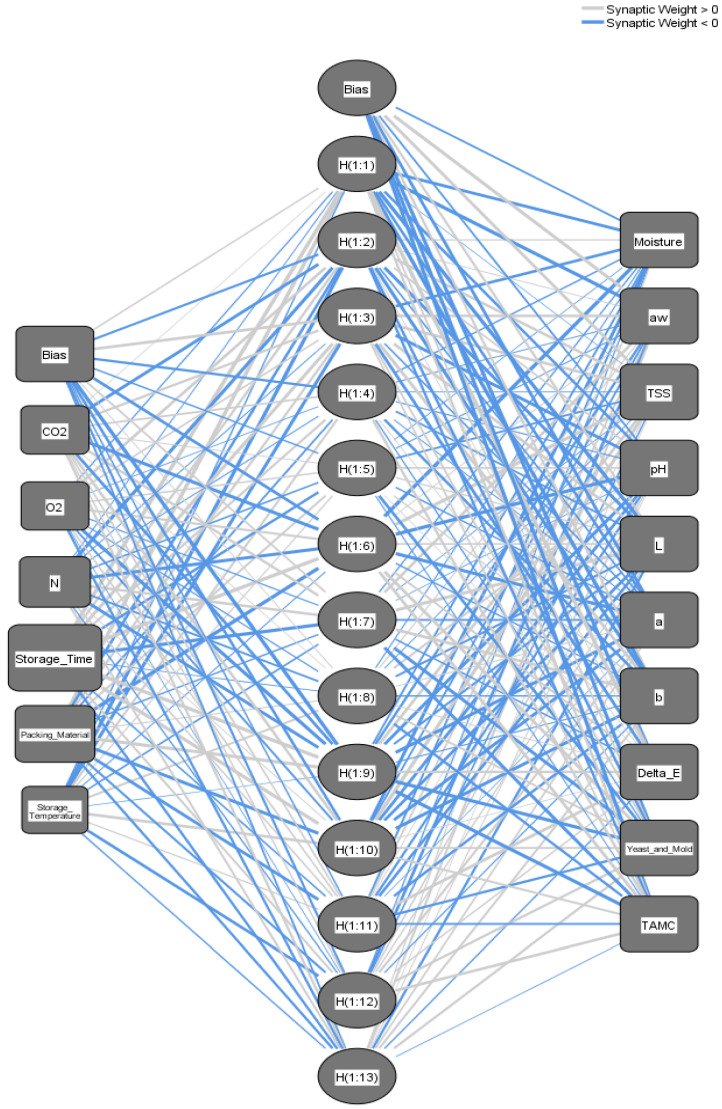
Networks architecture of the ANN prediction model for predicting the MC, aw, TSS, pH, L*, a*, b*, ∆E, yeast and mold count, and TAMC of the Khalas cultivar fruits during storage based on the storage condition parameters (storage time, storage temperature, packing material, N, O_2_, and CO_2_).

**Figure 10:**
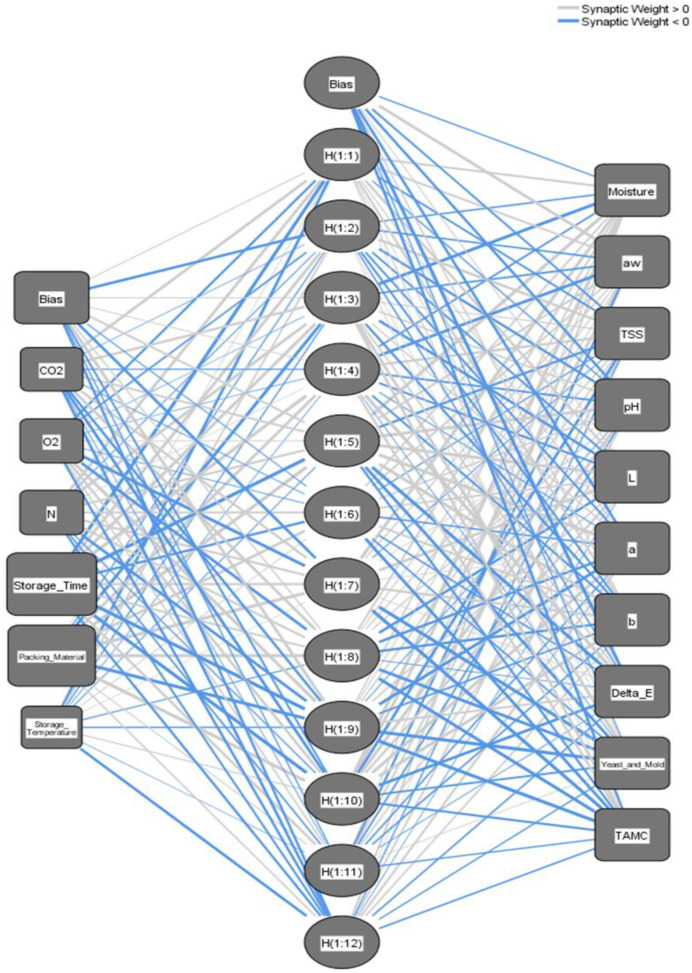
Networks architecture of the ANN prediction model for predicting the MC, aw, TSS, pH, L*, a*, b*, ∆E, yeast and mold count, and TAMC of the Sukary cultivar fruits during storage based on the storage condition parameters (storage time, storage temperature, packing material, N, O_2_, and CO_2_).

The ANN technique in SPSS was used to determine the prediction ANN model for each stored date fruit cultivar at the different storage conditions. The two developed ANN prediction models have one hidden layer. The input layer contained six nodes for the independent variables. The output layer contained 10 nodes for the dependent variables, as shown in Table 7. The rescaling method for covariates was normalized. The applied activation functions for the hidden layers were a hyperbolic tangent, and for the output layers was the Identity for all ANN prediction models at each stored date fruit cultivar under the different storage conditions. Overall, 60% of the dataset was used for training, 20% for model testing, and 20% for evaluation. The error function used was the sum of squares because of the Identity function. Figure 9 and Figure 10 show the optimal ANN architecture to predict the Khalas (Figure 9) and the Sukary (Figure 10) cultivars’ quality parameters during storage under different storage parameters. This architecture shows the six input nodes for storage conditions variables (storage time, storage temperature, packing material, N, O_2_, and CO_2_), the 13 hidden nodes for the Khalas cultivar and 12 for the Sukary cultivar, and the 10 output nodes describing the predicted values of the target quality parameters (MC, aw, TSS, pH, L*, a*, b*, ∆E, yeast and mold count, and TAMC). The trained ANN model efficiently predicted the target quality parameters when fed the system’s storage conditions variables data.

#### 3.4.2. Importance of the Independent Variable

**Figure 11:**
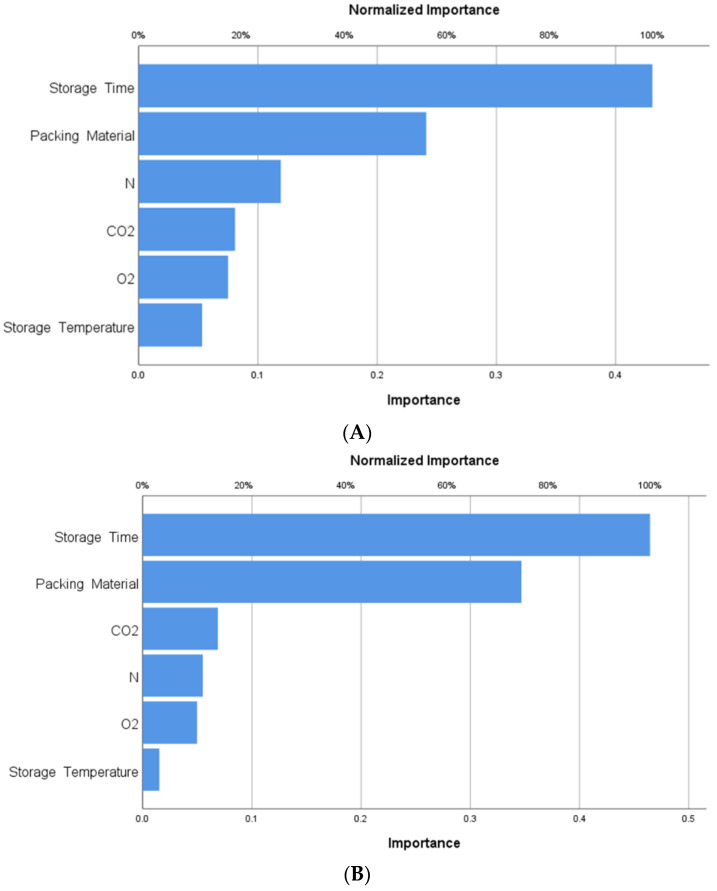
(**A**) Importance of the independent storge variable (storage time, storage temperature, packing material, N, O_2_, and CO_2_) for Khalas cultivar. (**B**) Importance of the independent storge variable (storage time, storage temperature, packing material, N, O_2_, and CO_2_) for Sukary cultivar.

**Figure 12:**
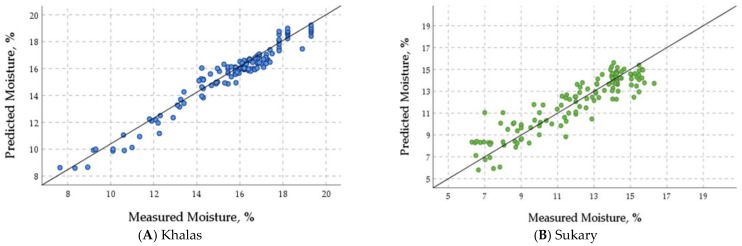

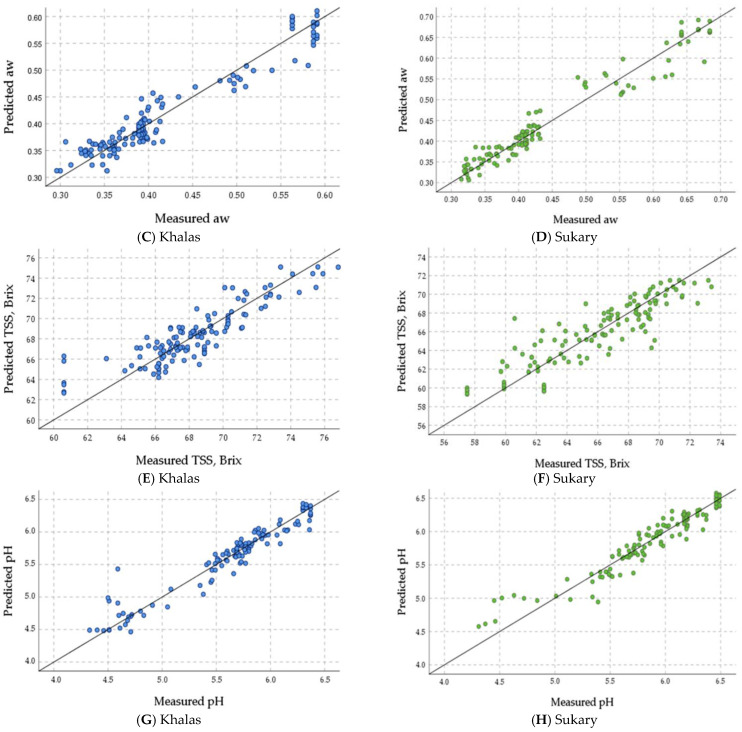
The plots of measured moisture content (**A**,**B**), water activity (**C**,**D**), total soluble solids (**E**,**F**), and pH (**G**,**H**) values of the stored fruits of cultivars Khalas (**A**,**C**,**E**,**G**) and Sukary (**B**,**D**,**F**,**H**) versus the predicted values by the ANN prediction models in the validation phase based on storage variables.

Figure 11 and Figure 12 show the importance of the independent variables (storage time, storage temperature, packing material, N, O_2_, and CO_2_) for Khalas and Sukary cultivars, respectively, in the ANN model regarding relative and normalized importance. These figures show the impact of the change of each storage variable on the ANN prediction model. The variables related to storage time and packing material have an essential effect on how the networks predict the dependent variables’ values (MC, aw, TSS, pH, L*, a*, b*, ∆E, yeast and mold count, and TAMC).

#### 3.4.3. Evaluation of the ANN Prediction Models

Table 8 compares the error outcomes of the ANN models (the sum of squares error, average overall relative error, and relative error) in the training, testing, and evaluating phases for cultivars Khalas and Sukary. The relative errors were shown depending on the dependent variables of the MC, aw, TSS, pH, L*, a*, b*, ∆E, yeast and mold count, and TAMC measurement levels. It was noticed that the datasets gave suitable results regarding the model errors in the training, testing, and evaluating phases. Based on these findings, adopting the independent variables (storage time, storage temperature, packing material, N, O_2_, and CO_2_) for Khalas and Sukary cultivars can efficiently predict the MC, aw, TSS, pH, L*, a*, b*, ∆E, yeast and mold count, and TAMC of the stored date fruits during storage.

Table 9 presents the evaluation values for the prediction models based on ANN used to forecast quality parameters of date fruit cultivars. The evaluation uses two widely used metrics: Root Mean Square Error (RMSE) and Mean Absolute Percentage Error (MAPE). The evaluation values presented in Table 6 indicate that the prediction models based on ANN have performed well in predicting the quality parameters of Khalas and Sukary date fruit cultivars. The low RMSE and MAPE values suggest a high level of accuracy in the predictions, providing confidence in the reliability of these models for predicting the quality parameters of date fruit cultivars. For example, regarding MC, the RMSE values are 1.251 for Khalas and 1.142 for Sukary, with corresponding MAPE values of 6.321% and 7.913%, respectively. These low values suggest that the prediction models perform well in estimating the MC for both cultivars. Similarly, the RMSE and MAPE values are relatively low for other quality parameters for Khalas and Sukary cultivars. This indicates that the prediction models have demonstrated good performance in predicting these quality parameters for the date fruit cultivars.

#### 3.4.4. Validation of the ANN Prediction Models

Figure 12 shows the plots of the measured MC, aw, TSS, and pH values of the stored fruits of cultivars Khalas (Figure 12A,C,E,G) and Sukary (Figure 12B,D,F,H) versus the predicted values by the ANN prediction models in the validation phase based on the storage variables (storage time, storage temperature, packing material, N, O_2_, and CO_2_). These figures showed that the ANN prediction models efficiently predicted the quality attributes with linear regression nearly overlapping the 1:1 line (y = x + 0). The linear regression between the measured and the predicted values of the Khalas MC was y = 0.82 + 0.95 x with R^2^ = 0.951, aw was y = 0.03 + 0.92 x with R^2^ = 0.927, TSS was y = 15.91 + 0.77 with R^2^ = 0.766, pH was y = 0.43 + 0.93 x with R^2^ = 0.927. The linear regression between the measured and the predicted values of the Sukary MC was y = 2.26 + 0.81 x with R^2^ = 0.829, aw was y = 0.02 + 0.96 x with R^2^ = 0.957, TSS was y = 14.22 + 0.781 with R^2^ = 0.788, pH was y = 0.64 + 0.91 x with R^2^ = 0.911. The R^2^ values, ranging from 0.766 to 0.957, indicate a good to excellent fit of the regression lines to the data, suggesting that the ANN models predicted the quality attributes, as the data points in the graphs closely aligned with the 1:1 line. This indicates a satisfactory level of agreement between the predicted and measured values.

**Figure 13:**
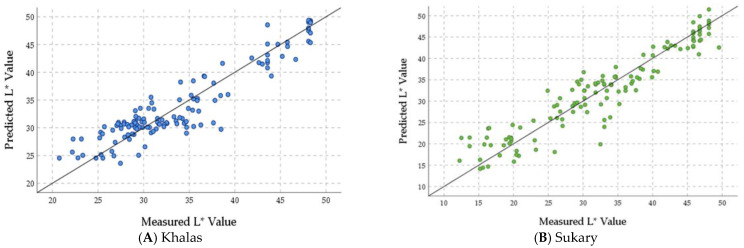

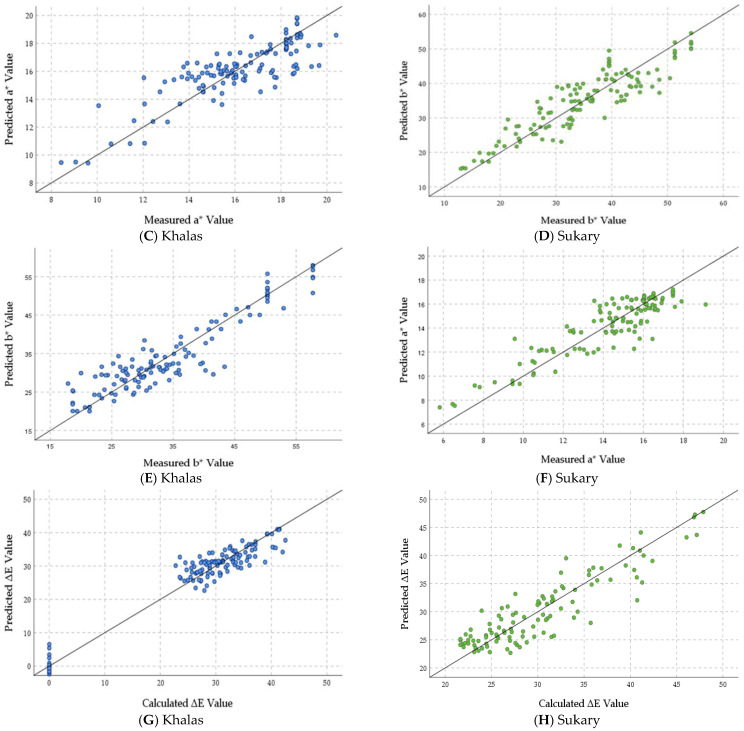
The plots of measured color parameters of L* (**A**,**B**), a* (**C**,**D**), b* (**E**,**F**), and ∆E (**G**,**H**) values of the stored fruits of cultivars Khalas (**A**,**C**,**E**,**G**) and Sukary (**B**,**D**,**F**,**H**) versus the predicted values by the ANN prediction models in the validation phase based on storage variables.

Figure 13 shows the plots of the measured L*, a*, b*, and ∆E values of the stored fruits of cultivars Khalas (Figure 13A,C,E,G) and Sukary (Figure 13B,D,F,H) versus the predicted values by the ANN prediction models in the validation phase based on the storage variables. These figures showed that the ANN prediction models efficiently predicted the color parameters with linear regression nearly overlapping the 1:1 line (y = x + 0). The linear regression between the measured and the predicted values of the Khalas L* was y = 4.65 + 0.886 x with R^2^ = 0.861, a* was y = 4.89 + 0.77 x with R^2^ = 0.692, b* was y = 5.13 + 0.85 with R^2^ = 0.854, ∆E was y = 1.67 + 0.44 x with R^2^ = 0.935. The linear regression between the measured and the predicted values of the Sukary L* was y = 3.89 + 0.88 x with R^2^ = 0.882, a* was y = 2.98 + 0.79 x with R^2^ = 0.805, b* was y = 6.85 + 0.81 with R^2^ = 0.813, ∆E was y = 1.53 + 0.94 x with R^2^ = 0.938. These results indicate that the ANN prediction models have shown high accuracy in estimating the color parameters of both Khalas and Sukary fruits during storage. The validation of the ANN prediction models’ performance reveals that they efficiently predicted the color parameters, and the predictions closely align with the measured values.

**Figure 14:**
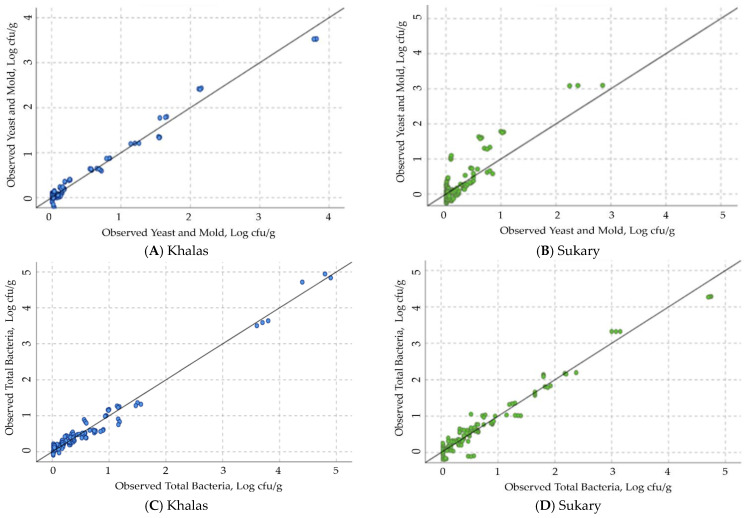
The plots of measured microbial load of yeast and mold (**A**,**B**) and total amount of microbial contamination (TAMC) (**C**,**D**) counts on the stored fruits of cultivars Khalas (**A**,**C**) and Sukary (**B**,**D**) versus the predicted values by the ANN prediction models in the validation phase based on storage variables.

Figure 14 shows the plots of the observed microbial load of yeast and mold (Figure 14A,B) and total amount of microbial contamination (TAMC) (Figure 14C,D) counts on the stored fruits of the cultivars Khalas (Figure 14A,C) and Sukary (Figure 14B,D) versus the predicted values by the ANN prediction models in the validation phase based on the storage variables. The linear regression between the observed and the predicted microbial load on Khalas with yeast and mold was y = 35.28 + 0.98 x with R^2^ = 0.980 and TAMC was y = 3.04 + 0.98 x with R^2^ = 0.979. The linear regression between the observed and the predicted microbial load on Sukary with yeast and mold was y = 1.3 + 0.81 x with R^2^ = 0.795 and TAMC was y = 5.19 + 0.96 x with R^2^ = 0.956. The predicted values were closely aligned with the observed values, as indicated by the term “linear regression nearly overlapping the 1:1 line (y = x + 0).” This suggests a strong correlation between the predicted and observed microbial loads. The ANN prediction models have demonstrated strong performance in estimating the microbial load of yeast and mold and the total microbial contamination (TAMC) on both Khalas and Sukary fruits during storage. The high R^2^ values suggest that the prediction models were reliable and accurate in forecasting microbial counts based on the given storage variables.

The findings of the current study indicated that using ANN regression models to predict the fruit quality of stored date fruits is a promising application of ANN, which can help improve the efficiency of producing and supplying perishable goods like date fruits. ANNs can model complex non-linear relationships between multiple interacting variables without requiring prior knowledge of the exact functional forms. This allows ANN to learn the underlying relationships directly from the experimental data through training, better handle ambiguous and incomplete datasets, and generalize to new predictions.

Mohammed et al. [27] developed ANN models to predict the physicochemical properties of date fruits during cold storage based on their electrical properties. The multiple linear regression (MLR) models were also developed and compared to ANN. They mentioned that the ANN demonstrated a high potential for efficiently predicting the physicochemical properties of date fruits during cold storage compared with MLR. ANN provided several advantages over traditional regression techniques for predicting physicochemical properties.

Additionally, unlike linear regression, which only describes single-variable relationships, ANNs can simultaneously consider all input variables to predict output quality parameters. These capabilities, along with the ANN ability to achieve higher predictive performance as evidenced by the higher R^2^ and lower RMSE values in this study, demonstrate that ANNs are a more powerful and suitable technique for establishing prediction models involving complex systems with unknown mathematical equations relating multiple variables, such as the relationships between the storage conditions and quality parameters of date fruits. Srinivasagan et al. [79] and Mohammed et al. [80] utilized ANN regression models to accurately predict and estimate the shelf life of stored date fruits. This application of NN models offers several advantages. The ANN models quickly analyzed the physicochemical attributes of the fruits using low-cost spectral sensors. They mentioned that the application of ANN models in this study demonstrated their potential to revolutionize food quality assessment and improve efficiency in producing and supplying perishable goods like date fruits.

## 4. Conclusions

The current study investigated the effects of various storage times and conditions on the quality attributes of dates at the Tamer stage. The quality attributes of the fruits were significantly affected by MAP gases of CO_2_, O_2_, and N, packaging materials, storage temperature, and storage time. The study revealed that MAP with 20% CO_2_ + 80% N at 4 °C for both cultivars had the optimal conditions to reduce color transformation and microbial growth during storage. In addition, the study utilized ANN prediction models to predict date quality attributes under different storage conditions. The RMSE and MAPE were used to evaluate the performance of the ANN models in predicting various quality attributes. The findings demonstrated that the developed ANN models accurately predicted the quality changes of packaged dates under different storage conditions with low error rates. Moreover, in the validation phase, ANN models displayed reliable performance in predicting various quality parameters, including moisture content, water activity, total soluble solids, pH, color parameters, yeast and mold count, and total microbial contamination. The validation process involved linear regression analysis, which confirmed the models’ reliability in predicting quality attributes on observed data. This indicated that ANN models can be utilized to develop optimal packaging and storage techniques. Based on this finding, the ANN models can be adopted to predict changes efficiently and accurately in the quality of packaged dates under various storage conditions, helping to maintain fruit quality, extend their shelf life, and ensure the delivery of high-quality products to consumers. This study contributes to reducing food waste and enhancing food safety by providing useful information on the impacts of storage conditions on date fruit quality and demonstrating the applicability of ANN models for predicting fruit quality attributes during storage. However, it is essential to acknowledge that additional datasets would be valuable to confirm the reliability and generalizability of the ANN models for a wider range of date fruit cultivars.

## Figures and Tables

**Table 1:** Characteristics of the tested packaging film.

Film Type	Material	Width (mm)	Thickness (µm)	WVTR (g/m^2^/24 h)	O_2_TR (cm^3^/m^2^/24 h)	CO_2_TR (cm^3^/m^2^/24 h)
High Barrier	PHB/PpHB	320	65	3.74	8.65	323.04

PpHB, PHB, WVTR, and TR refer to polypropylene high barrier, polyamide high barrier, water vapor transmission rate, and transmission rate, respectively.

**Table 2:** Treatment applied on date fruit cultivars under different packaging and storage temperature conditions for one year.

Fruit Cultivars	Packaging Material	Gas Concentrations			Storage Temperature	Treatment Codes
		N (%)	O_2_ (%)	CO_2_ (%)		
Khalas	Cardboard box	78.08 ± 0.05	20.95 ± 0.05	0.03 ± 0.01	24 ± 1 °C	KCaRT
					4 ± 0.5 °C	KCaCT
	MAP tray	78.08 ± 0.05	20.95 ± 0.05	0.03 ± 0.01	24 ± 1 °C	KNAPRT
					4 ± 0.5 °C	KNAPCT
		80 ± 0.05	0	20 ± 0.05	24 ± 1 °C	KMAPRT
					4 ± 0.5 °C	KMAPCT
Sukary	Cardboard box	78.08 ± 0.05	20.95 ± 0.05	0.03 ± 0.01	24 ± 1 °C	SCaRT
					4 ± 0.5 °C	SCaCT
	MPA tray	78.08 ± 0.05	20.95 ± 0.05	0.03 ± 0.01	24 ± 1 °C	SNAPRT
					4 ± 0.5 °C	SNAPCT
		80 ± 0.05	0	20 ± 0.05	24 ± 1 °C	SMAPRT
					4 ± 0.5 °C	SMAPCT

The values in refer to the initial concentrations ± standard deviation.

**Table 3:** Mean values of quality parameters of two date cultivars under different storage conditions.

Fruit Cultivars	Storage Conditions	Chemical Properties			
		MC	aw	TSS	pH
Khalas	KCaRT	14.14 ± 1.96 ^B^	0.41 ± 0.08 ^B^	62.95 ± 0.72 ^B^	5.78 ± 0.06 ^A^
	KCaCT	18.48 ± 0.42 ^A^	0.58 ± 0.03 ^A^	65.79 ± 1.67 ^A^	5.64 ± 0.13 ^B^
	KNAPRT	18.16 ± 0.39 ^A^	0.57 ± 0.03 ^A^	65.52 ± 1.55 ^A^	5.61 ± 0.14 ^B^
	KNAPCT	18.12 ± 0.34 ^A^	0.58 ± 0.04 ^A^	65.66 ± 1.33 ^A^	5.65 ± 0.12 ^B^
	KMAPRT	18.11 ± 0.33 ^A^	0.58 ± 0.03 ^A^	65.59 ± 1.92 ^A^	5.62 ± 0.15 ^B^
	KMAPCT	18.18 ± 0.31 ^A^	0.59 ± 0.04 ^A^	65.37 ± 1.88 ^A^	5.51 ± 0.18 ^C^
Sukary	SCaRT	9.64 ± 2.23 ^C^	0.42 ± 0.11 ^B^	64.92 ± 1.41 ^B^	5.79 ± 0.11 ^A^
	SCaCT	15.16 ± 0.45 ^A^	0.64 ± 0.03 ^A^	68.22 ± 1.39 ^A^	5.68 ± 0.16 ^AB^
	SNAPRT	14.57 ± 0.32 ^AB^	0.63 ± 0.03 ^A^	67.54 ± 1.52 ^A^	5.63 ± 0.22 ^BC^
	SNAPCT	14.73 ± 0.38 ^AB^	0.63 ± 0.04 ^A^	67.69 ± 1.32 ^A^	5.55 ± 0.23 ^BC^
	SMAPRT	14.49 ± 0.38 ^B^	0.62 ± 0.03 ^A^	67.81 ± 1.69 ^A^	5.49 ± 0.24 ^CD^
	SMAPCT	14.57 ± 0.37 ^AB^	0.63 ± 0.04 ^A^	67.66 ± 1.83 ^A^	5.42 ± 0.28 ^D^

The means (n = 252) with the same letters within each column for each date cultivar are not significantly different at *p* ≤ 0.05.

**Table 4:** Mean values of color parameters of two date cultivars under different storage conditions.

Fruit Cultivars	Storage Conditions	Color Parameters			
		L*	a*	b*	∆E*
Khalas	KCaRT	31.58 ± 7.2 ^B^	12.4 ± 3.39 ^B^	30.07 ± 9.79 ^C^	28.87 ± 12.75 ^A^
	KCaCT	33.94 ± 6.12 ^B^	13.44 ± 3.08 ^B^	32.38 ± 10.07 ^C^	25.51 ± 12.65 ^A^
	KNAPRT	40.76 ± 3.57 ^A^	16.34 ± 1.28 ^A^	38.77 ± 7.04 ^B^	17.8 ± 10.17 ^B^
	KNAPCT	42.18 ± 2.97 ^A^	16.31 ± 1.26 ^A^	40.05 ± 6.16 ^AB^	11.33 ± 6.32 ^C^
	KMAPRT	40.87 ± 3.7 ^A^	16.55 ± 1.2 ^A^	40.89 ± 5.61 ^AB^	11.49 ± 6.35 ^C^
	KMAPCT	42.43 ± 2.86 ^A^	16.82 ± 1.12 ^A^	43.84 ± 4.9 ^A^	9.11 ± 4.78 ^C^
Sukary	SCaRT	32.22 ± 6.75 ^D^	12.52 ± 2.62 ^B^	30.05 ± 7.87 ^C^	29.11 ± 13.08 ^A^
	SCaCT	34.13 ± 5.85 ^CD^	13.24 ± 2.02 ^B^	32.13 ± 7.36 ^C^	26.16 ± 11.54 ^AB^
	SNAPRT	35.75 ± 4.62 ^BC^	14.84 ± 1.49 ^A^	38.22 ± 4.58 ^B^	21.31 ± 10.08 ^B^
	SNAPCT	38.72 ± 4.26 ^B^	15.27 ± 1.23 ^A^	38.68 ± 5.22 ^B^	14.74 ± 6.51 ^C^
	SMAPRT	38.21 ± 3.63 ^B^	15.53 ± 1.17 ^A^	41.55 ± 3.98 ^AB^	13.96 ± 6.15 ^CD^
	SMAPCT	43.06 ± 2.55 ^A^	15.87 ± 0.97 ^A^	43.11 ± 2.79 ^A^	8.74 ± 4.31 ^D^

The means (n = 252) with the same letters within each column for each date cultivar are not significantly different at *p* ≤ 0.05.

**Table 5:** Mean values of microbial loads on two date cultivars under different storage conditions.

Fruit Cultivars	Storage Conditions	Microbial Load	
		Yeast and Molds (Log cfu/g)	Total Bacteria (Log cfu/g)
Khalas	KCaRT	1.66 ± 0.28 ^E^	1.24 ± 0.43 ^C^
	KCaCT	3.27 ± 0.72 ^A^	2.96 ± 0.85 ^A^
	KNAPRT	2.82 ± 0.52 ^B^	2.14 ± 0.58 ^B^
	KNAPCT	2.62 ± 0.43 ^BC^	2.01 ± 0.55 ^B^
	KMAPRT	2.46 ± 0.47 ^C^	2.01 ± 0.55 ^B^
	KMAPCT	2.16 ± 0.24 ^D^	2.03 ± 0.46 ^B^
Sukary	SCaRT	1.96 ± 0.33 ^C^	1.61 ± 0.44 ^C^
	SCaCT	2.84 ± 0.73 ^A^	2.19 ± 0.64 ^A^
	SNAPRT	2.51 ± 0.59 ^AB^	1.97 ± 0.56 ^AB^
	SNAPCT	2.37 ± 0.58 ^B^	1.83 ± 0.49 ^BC^
	SMAPRT	2.32 ± 0.49 ^B^	1.73 ± 0.46 ^BC^
	SMAPCT	2.19 ± 0.41 ^BC^	2.07 ± 0.63 ^AB^

The means (n = 252) with the same letters within each column for each date cultivar are not significantly different at *p* ≤ 0.05.

**Table 6:** Correlation between storage parameters and stored fruit of Khalas and Sukary characteristics.

Fruit cv.	Fruit Characteristics	Storage Parameters				
		Storage Time	Storage Temperature	N	O_2_	CO_2_
Khalas	MC	−0.241 **	−0.207 *	0.350 **	−0.350 **	0.350 **
	aw	−0.315 **	−0.051	0.111	−0.111	0.111
	TSS	0.324 **	0.022	−0.170	0.170	−0.170
	pH	−0.301 **	−0.127	0.285 **	−0.285 **	0.285 **
	L*	−0.321 **	−0.162	0.298 **	−0.298 **	0.298 **
	A*	−0.415 **	−0.054	0.172	−0.172	0.172
	B*	−0.312 **	−0.159	0.296 **	−0.296 **	0.296 **
	∆E	0.354 **	0.059	−0.125	0.125	−0.125
	Y&MC	0.105	0.102	−0.249 **	0.249 **	−0.249 **
	TAMC	0.134	0.098	−0.271 **	0.271 **	−0.271 **
Sukary	MC	−0.497 **	−0.184 *	0.437 **	−0.437 **	0.437 **
	aw	−0.347 **	−0.095	0.191 *	−0.191 *	0.191 *
	TSS	0.265 **	0.067	−0.148	0.148	−0.148
	pH	−0.324 **	−0.155	0.259 **	−0.259 **	0.259 **
	L*	−0.314 **	−0.156	0.469 **	−0.469 **	0.469 **
	A*	−0.254 **	−0.151	0.302 **	−0.302 **	0.302 **
	B*	−0.321 **	−0.103	0.375 **	−0.375 **	0.375 **
	∆E	0.425 **	−0.007	−0.143	0.143	−0.143
	Y&MC	0.110	0.123	−0.226 *	0.226 *	−0.226 *
	TAMC	0.173	0.145	−0.326 **	0.326 **	−0.326 **

** Correlation is significant at the 0.01 level (2-tailed), and * correlation is significant at the 0.05 level (2-tailed).

**Table 7:** Networks information of the ANN prediction models.

Networks Information			
Input Layer	Covariates	1	CO_2_
		2	O_2_
		3	N
		4	Storage Time
		5	Packing Material
		6	Storage Temperature
	Number of Units (Excluding the bias unit)		6
	Rescaling Method for Covariates		Normalized
Hidden Layer(s)	Number of Hidden Layers		1
	Number of Units in Hidden Layer (Excluding the bias) unit		13 for Khalas 12 for Sukary
	Activation Function		Hyperbolic tangent
Output Layer	Dependent Variables	1	Moisture
		2	aw
		3	TSS
		4	pH
		5	L
		6	a
		7	b
		8	∆E
		9	Yeast and Mold
		10	TAMC
	Number of Units		10
	Rescaling Method for Scale Dependents		Standardized
	Activation Function		Identity
	Error Function		Sum of Squares

**Table 8:** A comparison between the prediction models’ errors in the training, testing, and holdout phases for cultivars Khalas and Sukary.

		Date Fruit Cultivars					
		Khalas			Sukary		
Phases		Training	Testing	Evaluating	Training	Testing	Evaluating
Sum of squares error		140.172	40.2319		134.158	41.473	
Average overall relative error		0.374	0.2914	0.4323	0.363	0.419	0.398
Relative error	MC	0.313	0.2455	0.3860	0.509	0.546	0.485
	aw	0.120	0.0636	0.2189	0.089	0.124	0.078
	TSS	0.401	0.4598	0.3655	0.241	0.309	0.277
	pH	0.234	0.1708	0.2610	0.197	0.235	0.226
	L*	0.217	0.1727	0.4759	0.225	0.202	0.423
	a*	0.516	0.2601	0.4362	0.371	0.556	0.324
	b*	0.268	0.1224	0.3361	0.294	0.567	0.331
	∆E	0.199	0.0716	0.3795	0.250	0.472	0.253
	Y&MC	0.753	0.6675	1.3610	0.837	3.862	0.895
	TAMC	0.716	0.6636	1.5660	0.613	0.749	0.745

**Table 9:** A comparison between evaluation values for ANN models to predict quality parameters of date fruit cultivars using two metrics of rote mean square error (RMSE) and mean absolute percentage error (MAPE).

Quality Parameters	Khalas		Sukary	
	RMSE	MAPE, %	RMSE	MAPE, %
MC	1.251	6.321	1.142	7.913
aw	0.023	4.332	0.035	4.824
TSS	1.696	2.151	1.787	1.911
pH	0.243	2.721	0.219	2.204
L*	2.707	6.712	4.555	9.214
a*	1.418	7.121	1.465	8.523
b*	2.263	6.132	4.947	10.202
∆E	2.996	8.374	4.141	9.413
YM	0.316	8.152	0.311	7.532
TB	0.271	8.243	0.287	10.309

## Data Availability

The data used to support the findings of this study can be made available by the corresponding author upon request.
